# The addition of a developmental factor, *unc-62*, to already long-lived worms increases lifespan and healthspan

**DOI:** 10.1242/bio.027433

**Published:** 2017-10-20

**Authors:** Dror Sagi

**Affiliations:** Departments of Genetics and Developmental Biology, Stanford University Medical Center, Stanford, CA 94305-5329, USA

**Keywords:** Aging, Bioengineering, *Caenorhabditis elegans*

## Abstract

Aging is a complex trait that is affected by multiple genetic pathways. A relatively unexplored approach is to manipulate multiple independent aging pathways simultaneously in order to observe their cumulative effect on lifespan. Here, we report the phenotypic characterization of a strain with changes in five aging pathways: (1) mitochondrial reactive oxygen species (ROS) production, (2) innate immunity, (3) stress response, (4) metabolic control and (5) developmental regulation in old age. The quintuply modified strain has a lifespan that is 160% longer than the transgenic control strain. Additionally, the quintuply modified strain maintains several physiological markers of aging for a longer time than the transgenic control. Our results support a modular approach as a general scheme to study how multiple pathways interact to achieve extreme longevity.

## Abstract (precis)

**Summary:** This work uses a modular approach to combine five genes together to build worms that are long lived and much healthier than control animals, without a significant reduction in fertility. This article has an associated First Person interview with the first author of the paper as part of the supplementary information.

## INTRODUCTION

Aging is a complex phenomenon influenced by multiple genetic pathways (Kenyon, 2010; López-Otín et al., 2013; Guarente, 2013). Aging has been particularly well-studied in *Caenorhabditis* *elegans*, where more than 100 genes have been identified that can extend lifespan (Hamilton et al., 2005; Samuelson et al., 2007; Curran and Ruvkun, 2007). Examples of pathways that influence *C. elegans* lifespan are: (1) control of cellular damage and redox state by mitochondrial activity, (2) protection from bacterial pathogenicity, (3) resistance to cellular stress, (4) caloric restriction and (5) aberrant expression of developmental control genes in old age (Budovskaya et al., 2008; Fontana et al., 2010; Sánchez-Blanco and Kim, 2011; Shore and Ruvkun, 2013; Bennett and Kaeberlein, 2014; Mann et al., 2016).

Previous studies have focused on genes and pathways one at a time in order to examine the underlying mechanisms for lifespan extension. Recently, we have investigated the effects of manipulating many of these pathways simultaneously by expressing four transgenes with longevity functions in a transgenic strain (Sagi and Kim, 2012). The first longevity transgene was zebrafish *ucp2*, which has mitochondrial uncoupling activity, a function that is absent from *C. elegans* (Pfeiffer et al., 2011). Expression of zebrafish *ucp2* extended lifespan by 40% compared to a transgenic control strain. Uncoupling allows protons to leak into mitochondria without producing adenosine triphosphate (ATP) (Brand, 2000), thus reducing inner membrane potential. A lower potential attenuates mitochondrial production of free radicals, which reduces free radicals damage accumulation during aging (Brand, 2000).

The second longevity gene was zebrafish lysozyme, *lyz*, which has an anti-bacterial function that is not found in *C. elegans* lysozymes (Callewaert and Michiels, 2010). A strain expressing zebrafish *lyz* had a lifespan 30% longer than the transgenic control. Worm lifespan is limited by mild pathogenic effects from *Escherichia* *coli*, which is used as a food source (Garsin et al., 2003; Sánchez-Blanco and Kim, 2011). Lysozymes degrade the bacterial cell wall and thus are key players against bacterial pathogens. Hence, introduction of a vertebrate lysozyme could extend lifespan by improving innate immunity via reduction of pathogenicity from *E. coli.*

The third longevity gene was *hsf-1*, which encodes heat-shock transcription factor that induces expression of many stress-resistance genes. Overexpression of *hsf-1* extended lifespan 35% compared to the transgenic control (Prahlad and Morimoto, 2009).

The fourth longevity gene was *aakg-2(sta2)*, which encodes the gamma subunit of adenosine monophosphate (AMP)-activated protein kinase. This is a regulatory signaling molecule that responds to low ATP/AMP ratios and plays a key role in stress response (Greer et al., 2007). The sta2 mutation in *aakg-2* is a gain-of-function mutation that causes the enzyme to be constitutively active. *C. elegans* strains expressing *aakg-2(sta2)* had lifespans that were 45% longer than transgenic controls.

A transgenic strain was generated that expressed all four longevity genes: *ucp2*, *lyz*, *hsf-1* and *aakg-2(sta2)*. This strain had a lifespan that was 130% longer than the transgenic control, which is roughly the sum of the effects from each longevity transgene expressed alone.

One goal of this work was to generate a worm strain with an extremely long lifespan (130% longer than wild-type lifespan). A second goal was to examine the interactions between the different aging genetic pathways (i.e. epistatic interactions). We compared the effect of expressing a transgene alone versus in combination with other transgenes. We could then determine whether the transgene had a smaller, similar or larger effect on longevity when expressed in combination with multiple longevity genes versus expressed alone. A third goal was to examine the relative trade-offs between beneficial effects on lifespan and detrimental effects (e.g. on viability and fertility) for each longevity transgene. By expressing four longevity transgenes in one strain, it might be that the negative effects from each transgene would outweigh the positive effects on lifespan, resulting in a strain with poor viability and fertility. A fourth goal was to utilize a bioengineering approach to extend lifespan by expressing genes derived from the zebrafish, *Danio rerio*, in *C. elegans*. *D. rerio* has a lifespan that is about 50 times longer than the lifespan of *C. elegans* (Spence et al., 2008), and we were able to extend *C. elegans* lifespan by expression several genes from *D. rerio* that do not even have orthologs in *C. elegans*.

Here, we extend our previous work by manipulating a developmental factor as a fifth component, namely knocking down the HOX co-factor *unc- 62* (Homothorax) in a strain expressing four longevity genes. RNAi against *unc-62* in a wild-type worm has previously been shown to significantly extend lifespan (45%). The mechanism of lifespan extension via *unc-62*(RNAi) involves reprogramming several developmental pathways (Van Nostrand et al., 2013). First, *unc-62*(RNAi) decreases the expression of yolk proteins (vitellogenins) that aggregate in the body cavity in old age, thus reducing protein aggregation in old worms. Second, *unc-62*(RNAi) results in a broad increase in expression of intestinal genes that typically decrease expression with age, presumably allowing prolonged somatic function in old age. Lastly, in old worms, the intestine shows increased expression of several neuronal genes normally expressed in young animals. Intestinal expression of some of these genes in old age may be detrimental. For example, increased expression of *ins-7* limits lifespan by repressing activity of the insulin pathway transcription factor DAF-16/FOXO in aged animals (Murphy et al., 2007). Thus, the developmental control gene *unc-62* limits lifespan by altering developmental processes that normally limit lifespan, and these processes are not related to damage or metabolism per se.

## RESULTS

### *Unc-62* synergizes with metabolic components to progressively extend lifespan

Previous work had shown that a transgenic *C. elegans* strain (SD1905) simultaneously expressing four types of longevity genes [*Ce hsf-1; Dr lyz; Ce aakg-2(sta2); Dr ucp2*] had an extended lifespan by 130% compared to *unc-119* transgenic controls (Sagi and Kim, 2012). We tested whether reducing the activity of *unc-62* by RNAi could extend the lifespan of this strain further. For simplicity, we refer to the transgenic strain expressing four longevity genes (SD1905) as the ‘aging quadruple’ and SD1905; *unc-62*(RNAi) as the ‘aging quintuple’, or ‘quintuple worms’ hereafter. We compared the lifespan of the aging quintuple worms to the lifespan of the aging quadruple strain. As additional controls, we determined the lifespan of strains expressing three longevity genes {either [*Ce aakg-2(sta2); Ce hsf-1; Dr lyz*] or [*Ce aakg-2(sta2); Dr ucp2; Dr lyz*]} and two longevity genes{either [*Ce hsf-1; Dr lyz*] or [*Ce aakg-2(sta2); Dr ucp2*]} (Sagi and Kim, 2012) (Fig. S1; Table 1).

**Table 1.**
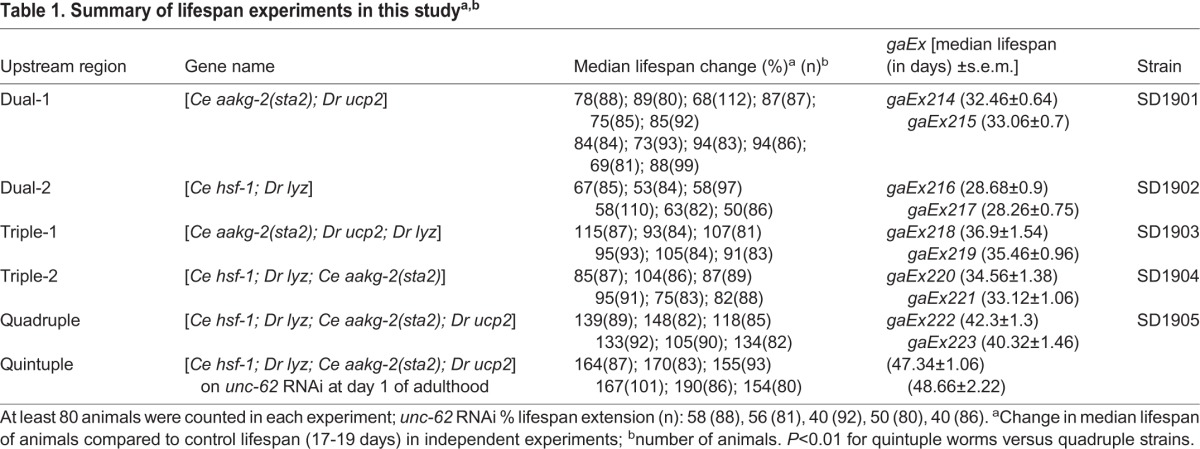
**Summary of lifespan experiments in this study^a,b^**

**Fig. 1.**
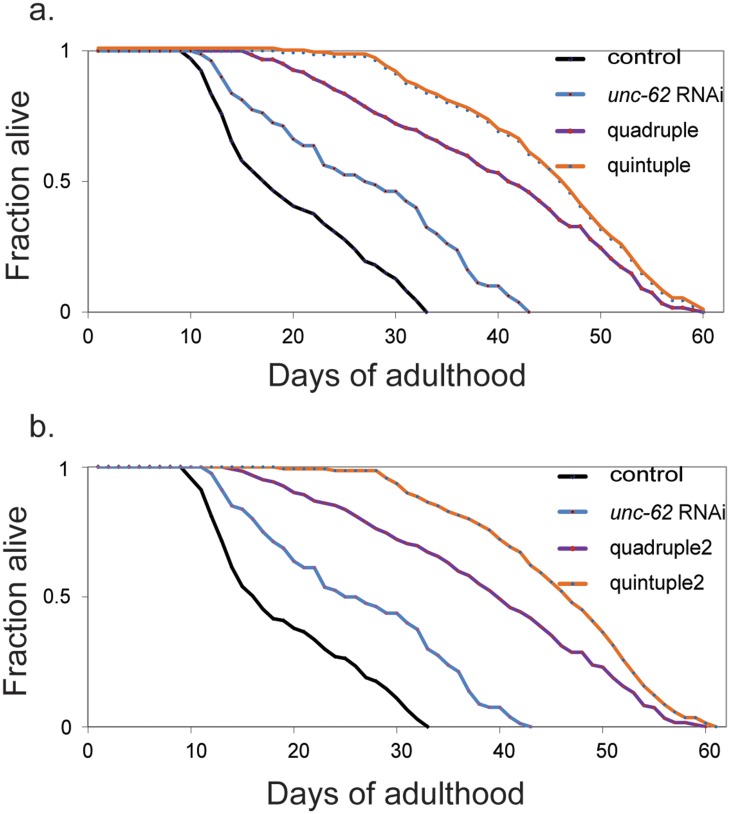
***unc-62* synergizes with quadruple** **lines to progressively extend lifespan.** (A,B) Representative lifespan curves of control, *unc-62* RNAi starting at day 1 of adulthood (60%, 56% extension), two independent quadruple lines (140%, 150%) and a quintuple strain (155%, 170%). The *x*-axis shows lifespan in days of adulthood. The *y*-axis shows fraction of worms alive. Control refers to transgenic worms expressing *unc-119(+); sod-3::mCherry*. Quintuple worms live 15% longer than quadruple strain (*P*=0.007, log-rank). Each experiment was repeated at least three times and representative data from one experiment is shown. Additional data are listed in Table 1.

*unc-62* activity was reduced by feeding bacteria containing double-stranded *unc-62* RNA to the aging quadruple strain beginning at day 1 of adulthood (Van Nostrand et al., 2013). Fig. 1 shows two lifespan assays, corresponding to each of the aging quadruple strains. The aging quintuple strain had a median lifespan of about 46 days compared to about 40 days for the quadruple strain and 17 days for the *unc-119* transgenic control. In both assays the median lifespan of the aging quintuple was 160% longer than that of the transgenic control (*P*<0.001; quintuple versus control, log-rank test), and 15% longer than that of the aging quadruple (*P*=0.007; quintuple versus quadruple, log-rank test). Table 1 shows data for four more lifespan assays, and each shows that the aging quintuple has a longer median lifespan than the aging quadruple. Using the complete data set of lifespan experiments comparing control, *unc-62* RNAi, quadruple and quintuple strains, we performed a two-way ANOVA analysis to compare the effects of *unc-62* RNAi in the control context as well as the quadruple. The analysis found that *unc-62* significantly extends the lifespan of the quadruple (*P*<0.001, two-way ANOVA).

### The aging quintuple has higher *sod-3::mCherry* expression, prolonged intestinal function and locomotory movement

In addition to lifespan, we examined several physiological markers of age to determine whether there was evidence that the health of the aging quintuple was extended compared to the aging quadruple. The first physiological marker of aging is expression of *sod-3::mCherry* in mid-age adults. Expression of *sod-3* decreases during aging, beginning at day 6 of adulthood (Budovskaya et al., 2008). The level of expression of *sod-3* in individual worms at day 9 of adulthood correlates with their individual lifespan; i.e. for worms at day 9, individuals with high expression of *sod-3* tended to live longer than individuals with low expression (Sánchez-Blanco and Kim, 2011). *sod-3* is a direct target of the DAF-16 FOXO transcription factor, which has a strong beneficial effect on lifespan (Kenyon, 2010). Hence a high level of *sod-3* in a given worm would indicate high levels of DAF-16 activity in that worm, leading to longer lifespan.

**Fig. 2.**
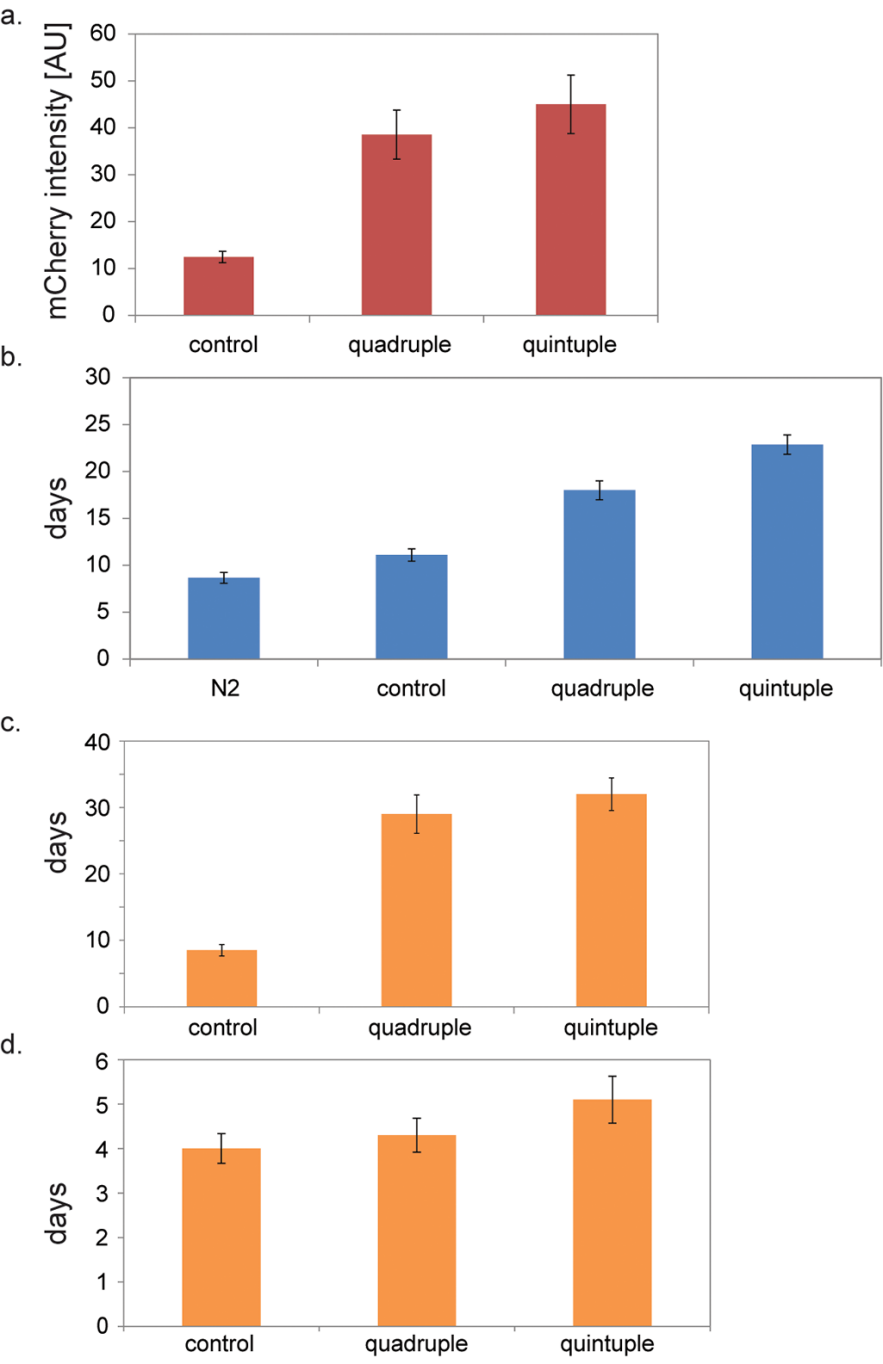
**Assays showing functional activity** **of physiological aging markers.** (A) Expression of *sod-3::mCherry*. Quadruple and quintuple worms show higher expression of *sod-3::mCherry* versus controls (*P*<0.001, *t*-test). The *y*-axis show fluorescence in arbitrary units. Error bars are s.e.m. of three independent experiments. (B) Intestinal clearance. Worms were grown on GFP-expressing bacteria. Fluorescence images of the intestine before and after 1 h transition to empty plates were analyzed every day to measure clearance of the GFP-labeled bacteria from the intestine. Quadruple and quintuple worms show prolonged intestinal clearance versus control or wild-type worms (*P*<0.001, *t*-test). In addition, aging quintuple worms have prolonged intestinal clearance than quadruple worms (*P*=0.003, *t*-test). The *y*-axis show days of complete intestinal clearance, starting from day 1 of adulthood. Error bars are s.e.m. of three independent experiments. (C) Days of free moving animals. Worms were monitored daily to assay declining in locomotor activity, manifested as the emergence of non-free moving animals (class B or class C). Quadruple and quintuple worms remain as free moving animals (class A) more than threefold longer than controls (*P*<0.001, *t*-test). The *y*-axis show days of adulthood as class A. Error bars are s.e.m. of three independent experiments. (D) Days as motionless animals (class C). All strains remain as class C for about 4 days. The *y*-axis show days as class C. Error bars are s.e.m. of three independent experiments.

To assay levels of *sod-3* expression, the fluorescence intensity of *sod-3::mCherry* was measured at day 13 of adulthood. Fig. 2A shows that the expression level of *sod-3::mCherry* was 360% higher in the aging quintuple than the transgenic control (*P*<0.001) and 15% higher than the aging quadruple (*P*=0.2). Since the maximum lifespan of the aging quintuple is about 60 days, this result indicates that *unc-62*(RNAi) has an effect in the aging quintuple strain relatively early in life (∼20% of maximum lifespan). While both the quadruple and quintuple strains have much higher *sod-3::mCherry* expression, *unc-62* does not further increase *sod-3::mCherry* levels beyond the quadruple levels.

The second physiological marker of aging is digestion of bacteria. Young worms are able to digest bacteria by grinding them in their pharynx and digesting them in their intestines. In old worms, some bacteria are not ground in the pharynx and remain intact in the intestine, where they can be visualized using fluorescently labelled *E. coli* (Greer et al., 2007). Worms were grown on *E. coli* expressing GFP and then moved to empty plates for one hour. Fluorescence images of the intestine before and after the transition to empty plates were analyzed to measure clearance of the GFP within intact bacteria from the intestine. The assay was repeated every day in an aging time course, and the day when 50% of the worms could not entirely clear their intestine was noted. Fig. 2B demonstrates that transgenic aging quintuple worms retain the ability to clear their intestine for 23 days, aging quadruple worms clear their intestine for 18 days, and transgenic control worms can clear their intestine for 11 days. Thus, the length of time that the aging quintuple can digest fluorescently labeled bacteria is 27% longer than that of the aging quadruple (*P*=0.003) and 100% longer than that of the transgenic control (*P*<0.001). The bacterial digestion assay was repeated three times with 30 worms for the aging quintuple [SD1905; *unc-62*(RNAi)], the aging quadruple strain (SD1905) and the transgenic control strain (SD1827), and yielded consistent results each time. We further used multiple *t*-test to validate that *unc-62* specifically extends intestinal clearance (*P*=0.0085). Fig. S2 shows a time course of one repeat, demonstrating the different kinetics of intestinal clearance for control, quadruple and quintuple worms.

The third physiological marker of aging is locomotory behavior. Worm movement can be classified into three groups (Herndon et al., 2002). Class A worms move freely and respond to prodding by vigorous movement. Class B worms do not move unless prodded and leave non-sinusoidal tracks. Class C worms are motionless and only twitch in response to touch. As worms age, they change from class A, to B and finally to C before they die. Essentially 100% of young control worms are class A. During aging, class B animals first appear at day 9 of adulthood, which is ∼50% of their lifespan. Class C animals first appear at about day 12 of adulthood. We used a ratio of 10% of class B worms in the population as a threshold for the appearance of class B worms (see Materials and Methods).

We assayed decline in locomotor activity during aging for the aging quintuple, the aging quadruple and the transgenic control strain (see Materials and Methods). For aging quintuple worms, class B animals first appeared after 32 days, about 70% of their median lifespan (Fig. 2C). For the aging quadruple strain, the majority of worms were class A until day 29, which is also about 70% of their median lifespan (Fig. 2C). These observations indicate that the aging quintuple not only remained active for a longer time than the controls, but that they were also mobile for a larger fraction of their lives.

A second way to measure the effect of aging on mobility is to note the length of time that class C persists during a lifespan. Fig. 2D shows that the time when most worms in the population were class C was about four days for all three strains. This indicates that the longevity genes in the aging quadruple strain and aging quintuple worms did not significantly extend the end of life, when worms are immobile and barely functional.

**Fig. 3.**
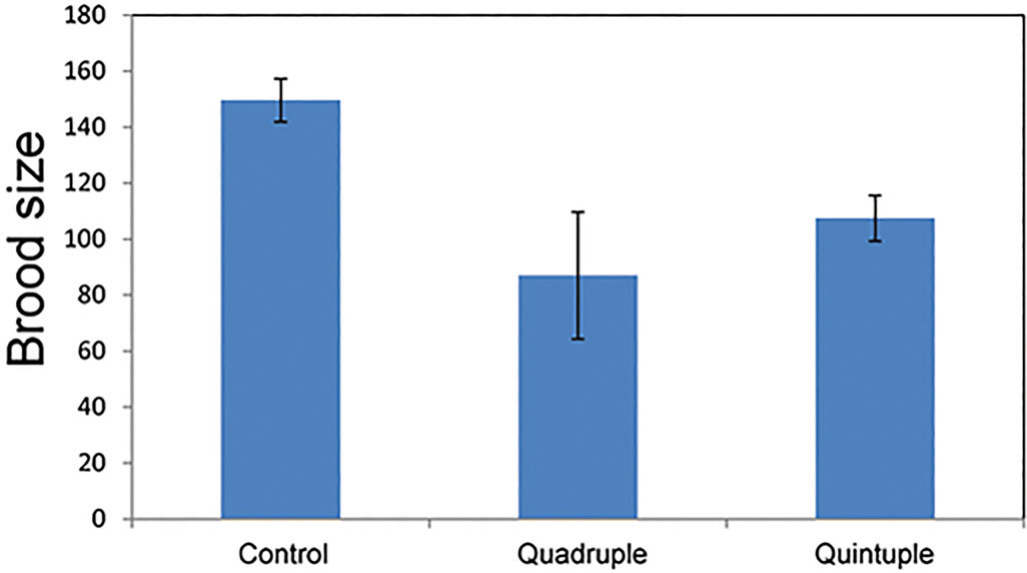
**Analysis of brood size.** Brood size was determined by counting the total number of progeny from a single hermaphrodite. The average and s.e.m. for eight animals is shown. The *y*-axis shows the total number of progeny from individual hermaphrodites. Control refers to worms expressing *unc-119(+); sod-3:mCherry*. The control bar represents an average of three independent lines.

We examined the brood size for the aging quintuple worms. One theory of aging posits that there is a balance between lifespan and fertility, and that the mechanistic explanation involves a trade-off of limited metabolic resources that are allocated either to germline maintenance resulting in fertility or somatic maintenance resulting in longevity. We examined whether the extended longevity exhibited by the aging quintuple worms came at the expense of fertility. We counted the number of viable offspring produced by the aging quintuple, the aging quadruple and the *unc-119* transgenic control strains (Fig. 3). The brood sizes of the aging quintuple and aging quadruple were 70% and 60% of the brood size for the transgenic control strain (*P*<0.01). The addition of *unc-62*, however, did not significantly affected the quadruple brood size (*P*=0.1, *t*-test). The decrease in brood size for the two longevity strains supports the germline-soma trade-off model for aging. However, the reduction in brood size is not as much as the increase in longevity.

## DISCUSSION

We describe a *C. elegans* strain termed an aging quintuple with a lifespan that is 160% longer than that of a transgenic control. The strain was constructed by manipulating five pathways involved in longevity: (1) reducing cellular reactive oxygen species via expression of a vertebrate mitochondrial function, (2) reduction of bacterial pathogenicity lysozyme by expression of a vertebrate lysozyme, (3) amplified stress response by expression of *hsf-1*, (4) activation of metabolic pathways by expression of an activated form of *aakg-2* and (5) reduction of activity of a developmental regulator *unc-62*. The first four pathways were modified by expressing a transgene in the aging quintuple that could activate the respective pathway (Sagi and Kim, 2012). The fifth pathway involved reducing *unc-62* activity by RNAi in adults. The aging quadruple strain, containing transgenes involved in the first four pathways, has a lifespan 130% longer than transgenic controls. The aging quintuple strain, in which all five pathways are altered, has a lifespan that is 160% longer than the transgenic control (Fig. 1).

These pathways extend lifespan by diverse mechanisms. The aging quadruple strain containing transgenes involved in the first four pathways has been described previously. Briefly, these four pathways are involved in cell damage, stress response and altered cell metabolism. The mechanism of lifespan extension from the fifth pathway involving *unc-62* is distinct from the other longevity pathways (Van Nostrand et al., 2013). *unc-62*(RNAi) has at least three beneficial effects for lifespan. The first is that *unc-62*(RNAi) prevents the accumulation of yolk proteins in old worms, after the cessation of reproduction. The second is that there is overall higher levels of gene expression in the intestine in *unc-62*(RNAi) than in transgenic controls, possibly related to cessation of transcription of the yolk protein genes that constitute a significant fraction of new RNA synthesis in the intestine. The third is that *unc-62*(+) is involved in turning on several neuronal genes in the intestine in old age, such as the *ins-7* which encodes an insulin-like growth factor. Expression of these neuronal genes in the intestine in old age is detrimental and shortens lifespan. *unc-62*(RNAi) prevents the late-life expression of neuronal genes in the intestine.

The aging quintuple strain also retains several physiological functions for longer times than transgenic controls, i.e. the healthy portion of life was extended as well as lifespan. *sod-3* is directly activated by the DAF-16 FOXO transcription factor that acts at the end of the insulin-like signaling pathway (Murphy et al., 2003). Activation of DAF-16 has many beneficial effects on *C. elegans* lifespan including activation of stress-response genes and increased levels of innate immunity (Kenyon, 2010). *sod-3* expression is a biomarker for the level of activity of DAF-16 and consequently correlates with the true physiological age (Sánchez-Blanco and Kim, 2011). The aging quintuple showed higher levels of *sod-3::mCherry* expression than transgenic controls (Fig. 2A), indicating higher levels of activation of DAF-16 and better overall conditioning.

The ability of worms to move declines during normal aging, and aging quintuple can move up through day 32, which is 250% longer than transgenic controls. Notably, the last portion of life when worms are unable to move was not extended in the aging quintuple (Fig. 2C,D). This suggests that lifespan extension was achieved by increasing the length of time when worms are relatively healthy (class A or class B movement) rather than the very end of life (class C).

The ability to digest bacteria declines during normal aging, which leads to an increase in bacterial pathogenicity in old age that limits lifespan (Garsin et al., 2003; Sánchez-Blanco and Kim, 2011; Shore and Ruvkun, 2013). The aging quintuple was able to clear fluorescently labeled *E. coli* for 23 days, which is about 100% longer than the transgenic control (Fig. 2B).

The aging quintuple strain also shows how multiple modules can compound to extend lifespan. *A priori*, it could be that adding more components to worms has diminishing returns after a set number, i.e. the aging quintuple may not live longer than the aging quadruple (diminishing returns). This result might be obtained if all of the pathways ultimately affected the same downstream network in which case activating more pathways may have limited additional benefit. This result might also signify that negative, pleiotropic effects from each pathway for viability offset the positive effect of the pathway for longevity. It could also be that by extending life beyond the normal lifespan of two weeks, an event or disease occurs that is not observed in a normal lifespan; by analogy, Alzheimer's disease was not observed in early human society because people did not generally live long enough to suffer from this disease. The pathways are known to alleviate deleterious events that occur in the first two weeks, but they may not be effective for the new disease or process that appears in extreme age.

Another possible result from the aging quintuple is that the effects of the longevity pathways in the quintuple could exceed the effects seen in worms with alterations in a single pathway (synergistic effects). As an analogy, curing one major human disease (i.e. cancer) may not significantly extend human lifespan because people would still succumb to the other major disease, such as heart disease or stroke. However, simultaneously curing three diseases (e.g. cancer, heart disease and stroke) may have a much larger effect on human lifespan than curing any one disease by itself.

Rather than diminishing returns or synergistic effects, each longevity pathway in the aging quintuple contributed to lifespan extension about the same as it did in single mutant worms. One possible interpretation of this result is that the longevity pathways in the aging quintuple are relatively independent. Another possibility is that the longevity genes may act on overlapping pathways, but that that effects from one gene alone may only have partial effects and that simultaneously modulating multiple genes at once can improve the effect on a longevity pathway. Since the lifespan of the aging quintuple is longer than that of the aging quadruple, it could be that adding or modulating additional longevity pathways would continue to show a beneficial effect on overall lifespan.

## MATERIALS AND METHODS

### *C. elegans* genetics

All *C. elegans* strains were maintained and handled as previously described (Brenner, 1974). 5-fluoro-2′-deoxyuridine (FUDR, Sigma) plates were made by supplementing nematode growth media (NGM) agar media with 30 µM of FUDR.

Genes used in this study were amplified by PCR from N2 worm genomic DNA. Generation of constructs containing zebrafish or human cDNA used worm upstream regulatory sequence as defined by the promoterome (Dupuy et al., 2004). If the required promoter was not part of promoterome, all intergenic sequence upstream of the gene of interest was used. cDNA of the gene of interest was obtained from Open Biosystems (Lafayette, CO, USA). The 3′ UTR was from the intron-containing *unc-54* gene.

Table 1 lists the strains used in this paper and the extent of their lifespan extension versus controls. The transgenic strains containing two, three or four longevity genes and *sod-3::mCherry* (SD1905) were previously constructed by Sagi and Kim (2012). This strain was made by microinjecting *unc-119(ed3)* worms with the longevity gene of interest at 10 ng/µl and PD4H1 [*unc119(+); sod3::mCherry*] at 80 ng/µl (Sagi and Kim, 2012). *sod-3::mCherry* is a reporter for *daf-16* activity.

### *unc-62* RNAi experiments

*unc-62* RNAi clones used were obtained from the Ahringer RNAi library as previously described (Kamath et al., 2003; Van Nostrand et al., 2013). RNAi knockdown experiments were performed on NGM plates supplemented with 30 µM FUDR, 100 µg/ml Ampicillin, and 2 mM IPTG to induce dsRNA expression. RNAi was initiated at day 1 of adulthood.

### Analysis of lifespan

Lifespan analyses were conducted on FUDR plates at 20°C as previously described (Apfeld and Kenyon, 1999). At least 80 worms were used for each experiment. Age refers to days following adulthood, and *P* values were calculated using the log-rank (Mantel-Cox) method, or two-way ANOVA as specified in the text. Individuals were excluded from the analysis if their gonad was extruded or if they desiccated by crawling onto the edge of the plate. Three lifespan assays were performed for each of the quadruple lines. Fig. S1 represents one of three lifespan assays that compared 2, 3 and 4 gene combination to the quintuple.

### Quantification of *sod-3::mCherry* fluorescence

Fluorescence images of *sod-3::mCherry* were taken as described (Sánchez-Blanco and Kim, 2011). Briefly, 10 age-synchronized worms at day 13 of adulthood were transferred to 1 mM aldicarb-NGM plates for 2–3 h to induce paralysis (Mahoney et al., 2006). Worms were then photographed using a 20× lens on a Zeiss AxioPlan Fluorescent Microscope. Levels of mCherry expression (in the head and the first two pairs of intestinal cells) were analyzed using ImageJ (Sánchez-Blanco and Kim, 2011). For any given comparison, all pictures were taken on the same day with the same microscope settings. Results from three independent sets of 10 worms were used to calculate the average expression level and standard deviation.

### Quantification of bacterial ingestion using GFP

For each experiment, 30 worms were grown on OP50-1 carrying plasmid pFPV25, which expresses GFP under the control of rpsM. At each time point, worms were transferred to plates without bacteria for one hour, and then placed on agarose pads on slides with Levamisole (25 mM) to paralyze the worms. GFP levels from intact bacteria in the intestine were measured by fluorescence microscopy on a 20× lens on a Zeiss AxioPlan Fluorescent Microscope. Quantification of the fluorescence in each worm was performed using ImageJ. The number of worms displaying GFP in their pharynx and intestinal track was also scored.

### Analysis of locomotion type

Analysis of locomotion phenotype and subsequent categorizing to groups was done as described in Herndon et al. (2002). Class A worms move freely and respond to prodding by vigorous movement away from the touch stimulus. Class B worms do not move unless prodded and leave non-sinusoidal tracks. Class C worms are motionless and only twitch in response to touch. Overall, these results were consistent in three replicates of 50 age-synchronized animals. To avoid counting sporadic sick worms early in adulthood as class B or class C, we used a cutoff of 10% class B worms in a plate as a threshold for the appearance of class B worms. The number of worms in each class was measured each day until more than 50% of worms were class B or class C. The aging time course was repeated three times.

### Analysis of brood size

Brood size was determined by counting the total number of progeny from a single hermaphrodite, as previously described (Sagi and Kim, 2012).

## Supplementary Material

Supplementary information

First Person interview
