# Supplementary material for: The addition of a developmental factor, unc-62, to already long-lived worms increases lifespan and healthspan
Source: Biol Open. 2017 Oct 20;6(12):1796–801. doi: 10.1242/bio.027433 (PMC5769649; doi:10.1242/bio.027433)
Supplement: Supplementary information [file biolopen-6-027433-s1.pdf]

© 2017. Published by The Company of Biologists Ltd | Biology Open (2017): doi:10.1242/bio.027433: Supplementary information

FIRST PERSON

First person – Dror Sagi

First Person is a series of interviews with the first authors of a selection of papers published in Biology Open, helping early-career researchers promote themselves alongside their papers. Dror Sagi is first author on 'The addition of a developmental factor, *unc-62*, to already long-lived worms increases lifespan and healthspan', published in BiO. Dror is a postdoc in Oded Rechavi's lab in the Department of Neurobiology, Tel Aviv University, Israel, where he uses molecular tools to develop durable and long-lived animals as models in the study of aging, and in particular extending the healthy portion of life.

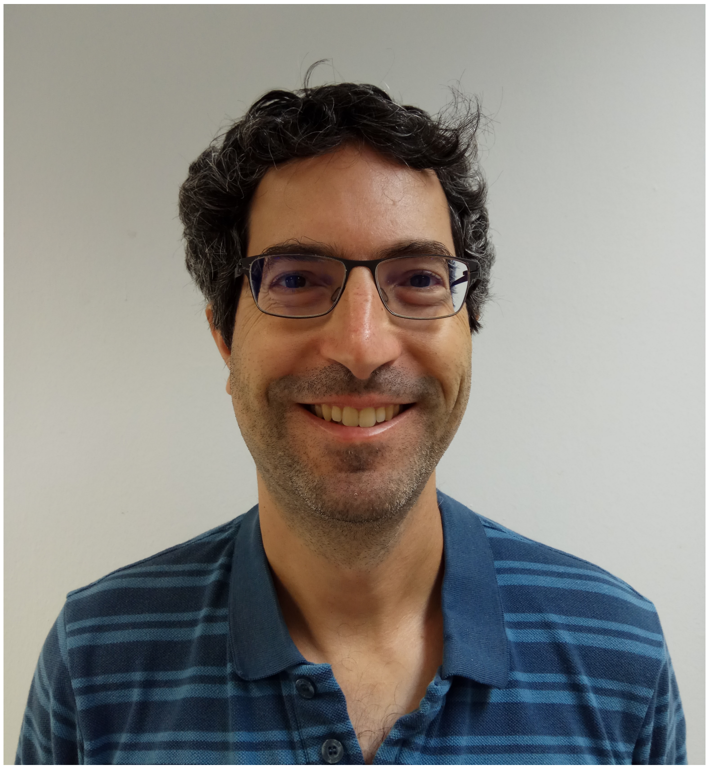

Dror Sagi

What is your scientific background and the general focus of your lab?

I have a PhD in physics, and did my postdoc on aging in the Department of Developmental Biology at Stanford University. My current lab focuses on different aspects of the RNA world in the nematode *Caenorhabditis elegans*. In particular the lab investigates transgenerational inheritance via small RNAs. That is, mechanisms that enable epigenetic inheritance and soma-to-germline transmission of information carried out by small RNA molecules.

How would you explain the main findings of your paper to non-scientific family and friends?

The paper shows that one can increase both lifespan and the healthy portion of life, healthspan, of the nematode worm *C. elegans*. Specifically, this paper shows that the genetically modified worms not only live longer, but also remain in much better condition for longer. Several key physiological features, such as locomotion, innate immunity and the digestive tract are much improved – when the regular worms become frail, immobile and can barely digest their food, the genetically engineered worms continue to move freely, eat well and are generally healthy.

"Animals do not age like machines due to wear and tear, but there is a genetic component that can regulate the rate of aging like any other biological process."

What are the potential implications of these results for your field of research?

This is a continuation of a previous work, and the combined message is that one can use an engineering approach to build durable animals using the same logic that we use today in human-made machines, such as cars. To achieve animals that are long-lived and healthy we used genetic manipulations of genes that are well studied, built on a solid understanding of their biochemical function as well as their role in aging. We consider these genes as genetic components. We then used a modular scheme to combine several components in the same animal based on their specifications, to improve biochemical functions that are associated with aging. Indeed, the worms were not only long-lived but were also much healthier. To summarize, this work suggests that 'rational design' could be used in a very complex phenotype, such as aging, to achieve longer lifespan and healthspan.

Dror Sagi's contact details: Department of Neurobiology, Tel Aviv University, Tel Aviv 6997801, Israel.

E-mail: dror.sagi@gmail.com

What has surprised you the most while conducting your research?

The magnitude of the effect. The genetically modified worms are much healthier. I did not expect that they would maintain such good condition for so long in comparison with the control.

What, in your opinion, are some of the greatest achievements in your field and how has this influenced your research?

For me, the two greatest achievements in the field were, firstly, the discovery that aging is under genetic control. Animals do not age like machines due to wear and tear, but there is a genetic component that can regulate the rate of aging like any other biological process. It started in worms, but has also been translated into other model animals, including mice. These studies started about 25 years ago with Cynthia Kenyon leading the way. Secondly, recent studies showing that longer lifespan could be epigenetically inherited. In particular, work in our lab has shown that even a Lamarckian stimulus, such as

Biology Open (2017): doi:10.1242/bio.027433: Supplementary information

short-term starvation, can induce longer lifespan for several generations. Epigenetically, inheritance of longer lifespan implies that two animals having very similar genomic sequences may vary in their lifespan due to ancestral history.

What changes do you think could improve the professional lives of early-career scientists?

Three things: 1) Mentoring by veteran faculty members; 2) reducing the amount of paperwork, which will free more time to focus on science; and 3) reducing the amount of teaching in the first couple of years. This would allow more focus on setting up a solid lab and getting grants.

What's next for you?

Hopefully, having my own lab.

Reference

Sagi, D. (2017). The addition of a developmental factor, *unc-62*, to already long-lived worms increases lifespan and healthspan. *Biol. Open* **6**, doi:10.1242/bio.027433.
